# Mumbai's business landscape: A spatial analytical approach to urbanisation

**DOI:** 10.1016/j.heliyon.2021.e07522

**Published:** 2021-07-09

**Authors:** Eric Vaz, Bruno Damásio, Fernando Bação, Mahender Kotha, Elissa Penfound, Shailendra Kumar Rai

**Affiliations:** a Department of Geography and Environmental Studies Toronto, Ryerson University, ON, Canada; b NOVA Information Management School (NOVA IMS), Universidade Nova de Lisboa, Lisboa, Portugal; c School of Earth, Ocean and Atmospheric Sciences, University of Goa, Goa, India; d Management Development Institute Gurgaon, India

**Keywords:** Data science, Self-organizing maps, Spatial regressions, GIS

## Abstract

India has proven to be one of the most diverse and dynamic economic regions in the world. Its industry focuses predominantly on the service sector and immediate economic growth seems to steer India into the economic superpower. India's unique business landscape is felt at a regional level, where massive urbanization has become an unavoidable consequence of population growth and spatial allocation to the economic hubs of metropolitan cities. Mumbai, one of the world's largest cities, represents a unique combination of a diverse economic landscape and the growth of a megacity. The role of Mumbai in India's growth is of crucial importance for India's business landscape. This paper explores the massive urbanization processes of Mumbai's peri-urban areas and compares urban sprawl with the location of its business landscape. A spatial accounting methodology based on the proximity of Mumbai's different economic hubs will be used to measure the underlying pattern of the Mumbai region, concerning past and present urbanization, and the effect of this urbanization process has on the possible location of businesses. This business-urban ecosystem perspective will be implemented by a spatial analysis on the correlation between urban compactness and urban footprints, in relation to business concentration and its spatiotemporal evolution over the last hundred years.

## 1 Introduction

India's economic prosperity flourished after 1991, mostly due to the liberalization of the Indian market. However, during the post-liberalization period, there was a harmful and robust tendency in the Indian corporate sector towards misallocation of resources (Kumar and Bhaduri, 2014). While India's economy grew significant speculation to India's economic growth over the last three decades and its future emerged (Ahluwalia 2005). As thus, despite India's successful performance in the last decades, India is facing times of uncertainty, far from a sustained economic transition, due to existing strains in the global economy (Mazumdar, 2014). India's economic growth must equate its allocation of resources, as well as the sustainability of its regional environment and human capital, to address possible future challenges on the fringe of its economic growth (Kuruvilla and Ranganathan, 2010). To mitigate probabilities of backlash of economic asymmetries, it must thus be addressed from a spatially-explicit level (Yue et al., 2014) to offer an integrative vision of India's sustainable economic and environmental future.

In the case of India, in detriment of the agriculture and rural world, economic urban hubs became ubiquitous signs of a modernized continent (Pandey and Seto, 2015). This has been accompanied by unprecedented change in the service sector, leading to a two trillion-dollar market at present. Mumbai, the financial capital of India, has witnessed as a result, an unparalleled change in its urban form, as well as its business landscape. The Greater Mumbai Area poses a unique opportunity to solidify India's economic growth but also brings several challenges relating to the environment (Vaz, 2014), rapid urbanization (Shafizadeh-Moghadam and Helbich, 2015; Sridhar et al., 2015) and life quality (Mathews, 2014). Economies of agglomeration must be well understood to equate at a geographical scale, the role of metropolitan areas, particularly within different industries (Rosenthal and Strange, 2003). Urbanization should be understood for India in context with its economic activity and land use, as entrepreneurship, and thus industry location, can have a significant role on future urbanization (Glaeser et al., 2015). The availability of spatial methods allows for an accurate perception of the dynamics of land use and business location simultaneously (Burrough, 1986). In this sense, smart urban and economic growth may be leveraged through spatial interactions and assessment of urban structures (Floater et al., 2014). Understanding the dynamics of urban land use may share important information on economic activity which may be spatially measured through the processes of agglomeration and urbanization (Zhou et al., 2015) Further, the geographical incorporation of suburban agglomeration, particularly in line with transportation optimum (Pucher et al., 2007), may depict a better perception of urbanization, linking economic efficiency and the business landscape (Vaz, 2013). The implementation of strategic foresight for India's economy becomes a consequence of the management of land use and imposes on the existence of economic activity utilizing the regional vertices of economic hubs, and the location of businesses and industry (Sahay and Mohan, 2003). To address such issues, spatial analysis and landscape metrics provide valid and useful tools to mitigate the impact over the environment, as well as to confer the potential of allocation of business in supporting rapidly growing regions. These techniques enable to.

(i) Foster land use policies for better local management by means of transportation efficiency (Waddell, 2011),
(ii) Relate to future environmental concerning in unplanned and disparate demographic regions (Piketty, 2014),
(iii) Create efficient support systems for optimizing business activity within urban regions and understand its spatial factors (Ghani et al., 2014).

Such a combinatory approach allows to test vulnerability at the regional level (Turner et al., 2003), which eventually leads to coupling Geographic Information Systems (GIS) to promote better decision-making (Nijkamp and Scholten, 1992; Malczewski and Rinner, 2015). Significant advances in the field of GIS relating to land use change and spatial system dynamics have brought increasing insights to (i) local processes, (ii) processes of diffusion and (iii) modified spatial structures, which may benefit from spatial analysis (Vaz, 2020; Neuwirth et al., 2015). Urban growth modelling has allowed a thorough empirical analysis of the consequences of urban change (Clarke et al., 1997; Barredo et al., 2003; Vaz et al., 2012; Arsanjani et al., 2013). The availability of novel algorithms allows to test the non-linear patterns of urban dynamics thanks to the advances in geocomputation (Liu et al., 2014a, b; Liu et al., 2014a, b; Vaz et al., 2021; Veerbeek et al., 2015; Vizzari and Sigura, 2015). Allied to geovisualization, these advances bring important tools for policy, enhancing the potential of non-linear simulation for decision makers (Kamateri et al., 2015). These non-linear approaches add the understanding the complexity of cities as engines of socio-economic growth and development (Vaz and Arasnjani, 2015). At country level, India's economic growth and financial development, is becoming the leading culprit of environmental degradation (Sehrawat et al., 2015). At the local level, in cities such as Mumbai, this is particularly significant and holds a strong geographical context, where the loss of biodiversity and environmental sustainability seems to be at stake due to rapid urbanisation and unplanned economic growth (Vaz, 2014).

An important issue that arises from the combination of rapid urbanisation, is the degree of pressure on the environment due to unplanned economic activity suggesting disparities at the regional level (Sachs et al., 2002). This paper addresses this combinatory issue through (i) a quantification of urban footprint in Mumbai, and testing Mumbai's urban form regarding its compactness and fragmentation, linking it to its (ii) agglomeration of businesses along the Greater Mumbai Area (GMA). In this sense, sprawl is not only measured in the direct relation to economic activity but also (iii) a sector-based assessment of the allocation of India's businesses becomes evident. Methodologically, the paper becomes a combination of spatial autocorrelation techniques with landscape metrics for urban land use, answering from a geographical perspective, the transitions of Mumbai's business landscape and its urban processes.

## 2 Study area

**Figure 1:**
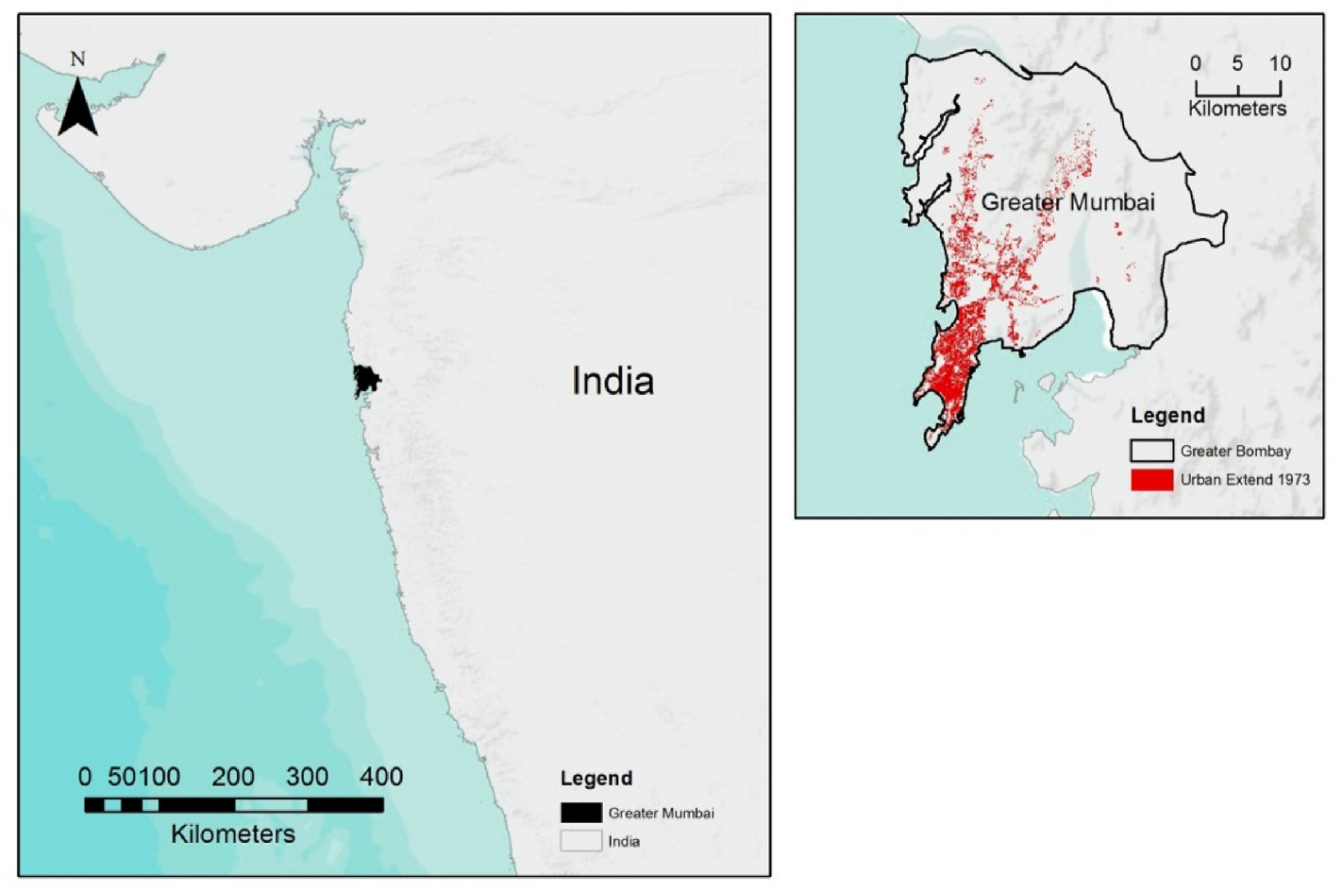
Location of study area.

The Mumbai Metropolitan Region (MMR) extends from 18° 53 to 19° 16 N and 72° to 72° 59 E (Figure 1).

As India's financial hub, it is one of the main economic regions of India, and is part of the western state of Maharashtra. As the fifth-largest metropolitan region in the world, it accounts for 12.5 million inhabitants based on its last census (2011). The Mumbai Metropolitan Region, such as most regions in Industrialized India face a series of challenges such as (i) air pollution (Kumar and Abba, 2006), (ii) urban growth (Shafizadeh-Moghadam and Helbich, 2015), (iii) coastal area (Murthy et al., 2001), (iv) poverty (Baker et al., 2005), and (v) environmental quality (Kamble and Vijay, 2011). The total urban area in Mumbai is 466.35 km^2^ with a maximum width of 17 km east to west and 42 km north to south. In its quest to become a world-leading economic hub, Mumbai must meet the imminent challenges brought by the global economy, while facing daunting social, economic and environmental issues within an ever-expanding urban core (Onilude and Vaz, 2021). The metropolitan area pertains one-tenth of India's factory and manufacturing employment (Swaminathan, 1995). Most of India's foreign trade passes through Mumbai, and with the ongoing liberalisation policies, integration of global markets tend to encourage the ongoing financial growth of the Mumbai Metropolitan Region, supporting India's business growth and dynamics. In this sense, the MMR is not only a city, but represents India's primary gateway to India's future economy, and must exemplify a modern vision of its integration towards sustainable allocation of businesses, and set an example for its many challenges for a sustainable future, particularly in line with its recent urban change (Phadke, 2014).

## 3 Data

**Figure 2:**
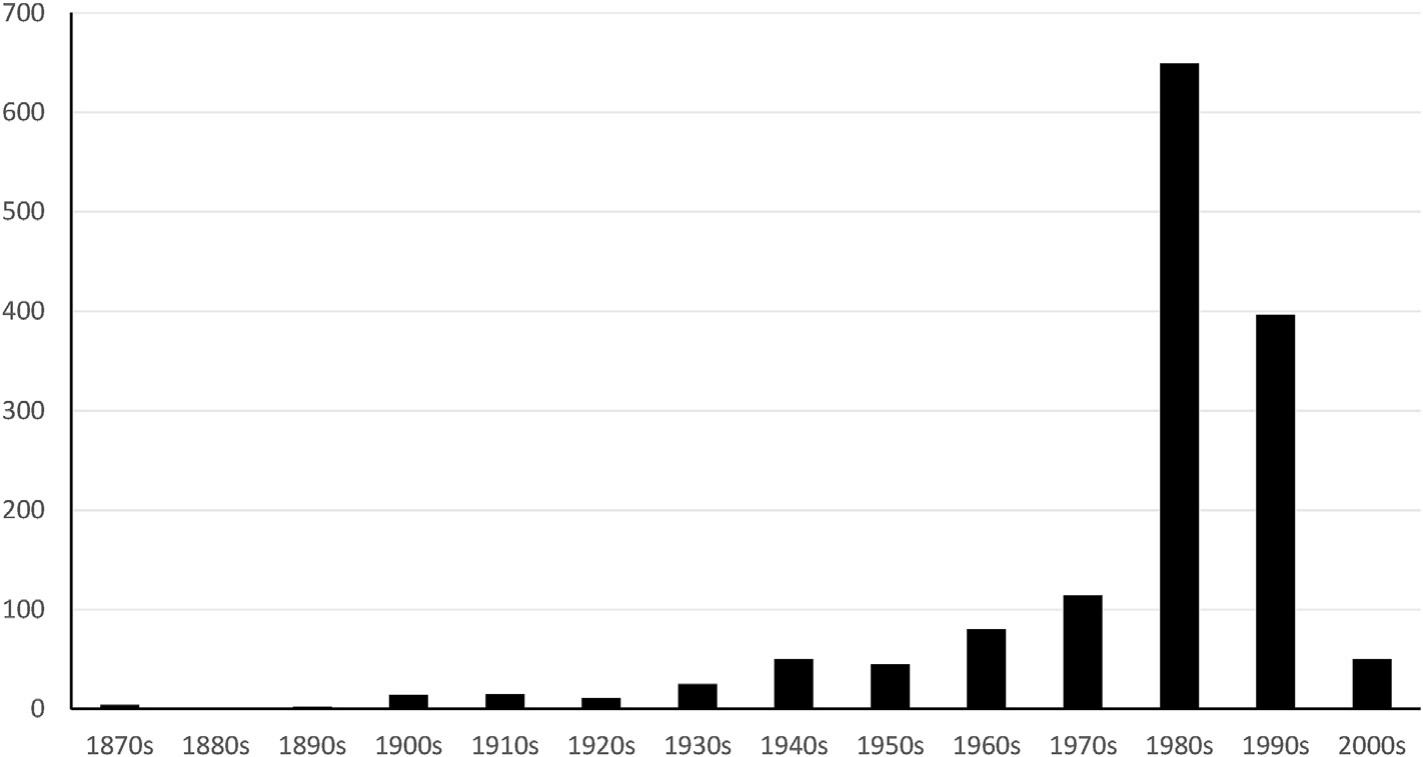
Industries in the Greater Mumbai Area by decade.

Postal addresses of Mumbai's registered industries were computed through geopy^1^. Misinterpreted or incomplete addresses were discarded. A final assessment to validate the classification of the business was carried out by means of a random selection of one 10% of the sample. These points were then projected onto Google Earth, and ancillary crowd sourced information as well as additional web search of addresses and businesses was conducted to certify attributes such as exact location, street number, and additional information of the location of business. This first step was the most demanding in terms of correct analysis and filtering of results, however, it was the least demanding in terms of computational power considering the total data set of a total of 1459 industries in the Greater Mumbai Area. Besides incorporating latitude and longitude values, the resulting data set also registers year of inception (ranging from 1863 to 2008; three industries did not report year of inception) and industry sector. The Figure below (Figure 2) shows the evolution of industries in Mumbai per decade.

Much with the present literature on India's business landscape, the eighties and nineties were of utmost important for Mumbai's economic growth. Since early 2000, a decrease in the registered industries in Mumbai has been witnessed. Further inspection of the topology of businesses allowed to summarize industrial sectors in Mumbai (Table 1). The dominant sectors of activity are linked to financial services, information technologies, chemicals, metals, and chemicals.

**Table 1:** Industrial sectors in Mumbai.

Industries	Total
Other financial services	184
Trading	172
Investment services	96
Computer software	78
Drugs & pharmaceuticals	65
Other asset financing services	43
Gems & jewelry	32
Textile processing	32
Commercial complexes	24
Metal products	21
Organic chemicals	21
Other chemicals	20
Steel	20

**Figure 3:**
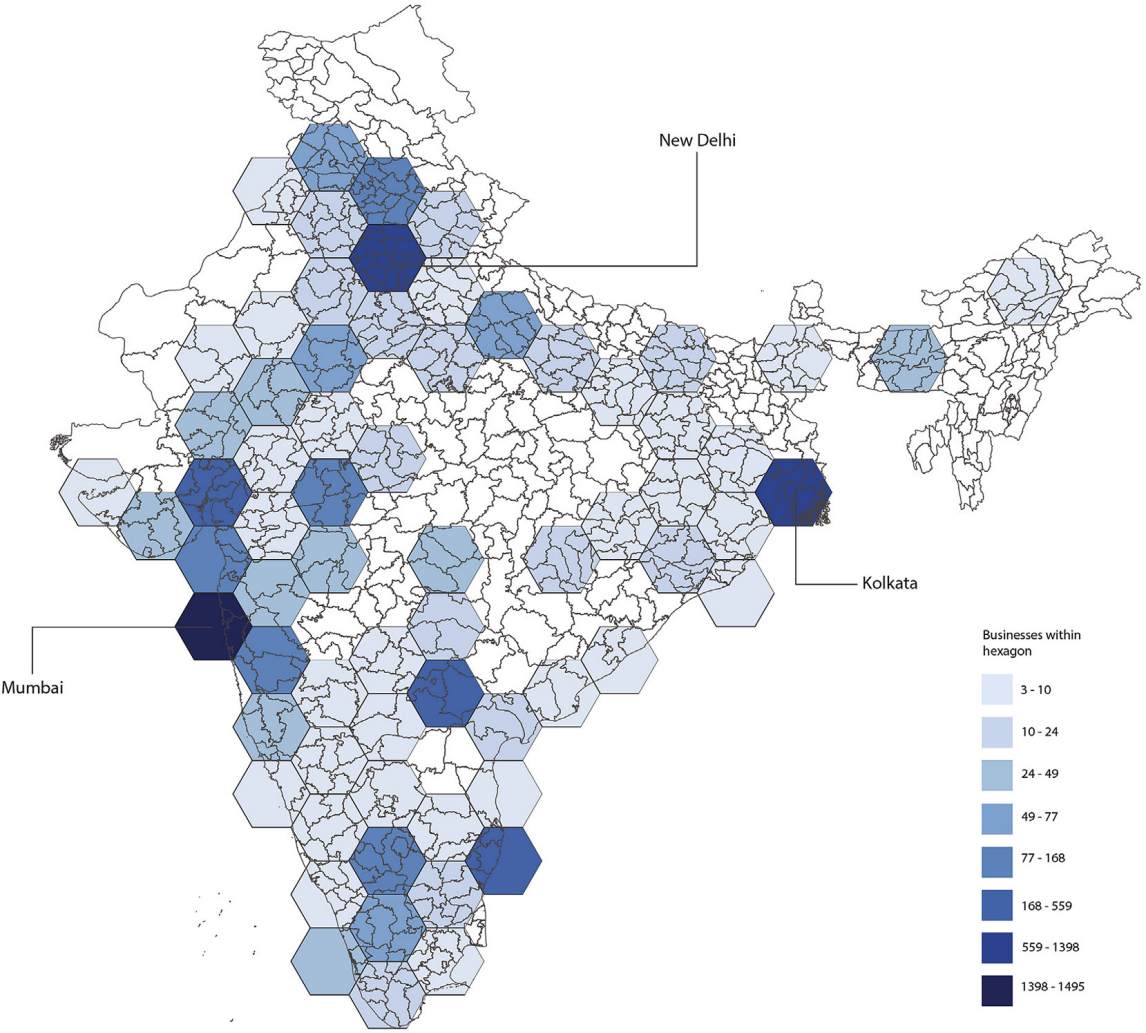
Hexagonal topology for India's industries.

The service sector has become dominant over the last decades in Mumbai; information technologies have mostly grown during the late-eighties. The spatial component of the distribution of Mumbai's business landscape was achieved by a correlation of urban land use with a hexagonal grid, which allowed to create a common topology for comparison of the different data sources. The figure below (Figure 3) represents the hexagonal topology that was implemented with an overview for India. The importance of Mumbai's industrial power becomes evident. Hexagons are particularly useful for comparing multi-layers as they avoid sampling bias from edge effects. Given the regional dimension of Mumbai's metropolitan area, distortion of curvature could be present, which becomes thus avoided by using a hexagonal grid.

The availability of Landsat imagery since the early seventies as well as mostly free availability as a data source, make this data source particularly efficient for regional land use classification, particularly for urban land use (Haack et al., 1987). Supervised classification was conducted for the Landsat imagery ranging over the different Landsat missions: Landsat MSS for the 1973 footprint, Landsat TM for 1990, Landsat ETM for 2001, and Landsat ETM + for 2010. For regional assessments of India's large megacities, the combination of Landsat imagery has proven to be effective (Taubenböck et al., 2009). Further geometric corrections were conducted and a maximum likelihood algorithm was used. A final kappa coefficient of over 80 percent was obtained for the urban land use covers, corresponding to a very positive result in its classification (Xiao et al., 2006).

## 4 Methodology

An integrated spatial analysis for business was conducted based on a combinatory approach of land use analysis and spatial autocorrelation metrics. At a first instance, the existence of spatial autocorrelation was tested at global level, as to understand whether Mumbai in fact, represents a spatial cluster, and if so, for which industries are these clusters mostly evident. This was carried out by determination of a global Moran's I test (Moran, 1950) for the Mumbai Metropolitan Region. This test offers useful insights on the global spatial autocorrelation of a predetermined geographical space. The Moran's I value of +1 indicates that features are clustered and there is a positive autocorrelation; however, a Moran's I value of -1 indicates that features are dispersed and there is a negative spatial autocorrelation. Moran's I value (Equation 1) of 0 indicates that features are distributed randomly, and therefore, spatial autocorrelation would not exist. Moran's I value can be computed as,

(1) $$I=\;\frac{n}{S_{0}}\frac{\sum_{i=1}^{n}\sum_{j=1}^{n}w_{i,j}z_{i}z_{j}}{\sum_{i=1}^{n}z_{i}^{2}}$$

where $z_{i}$ is the deviation of an attribute for feature I from its mean ($x_{i}-X)$, $w_{i,j}$ is the spatial weight (Equation 2) between features *i* and *j*, and *n* is the total number of existing features. As such, $S_{0}$ is the aggregate of all the spatial weights, which is calculated as follows.

(2) $$S_{0}=\sum_{i=1}^{n}\sum_{j=1}^{n}w_{i,j}$$

The demonstration of spatial autocorrelation at global level allowed proceeding with a local analysis of identifying business hotspots at local level. This was done by calculating the Local Gi∗ statistic and adopted to express business density per hotspots. This was conducted by a count function per Indian parish (administrative level 3). The count function expressed a business landscape, which allowed determining the spatial-autocorrelation of hotspots for Mumbai (Getis and Ord, 1992). The dataset contains temporal specific information on the creation of the business, as well as economic activity. The geocoding process consisted of identifying the total number of businesses for India, and rectifying when necessary by comparing local addresses to guarantee the accuracy of business location. A total of 1459 businesses were correctly classified for the Greater Mumbai Area, and accuracy performance tested by means of integrating Google Maps API for validating existing addresses. Further to this, a spatial index was created, allowing not only to an aggregate location within the Greater Mumbai Area, but also an interpretation of business typology. The North American Industry Classification System (NAICS) was adopted as a general classification schema, guaranteeing an adequate nomenclature for business and industry typologies. The NAICS is the standard used at the Federal level for classifying business establishments and North American statistical data. Adopted in 1997, it is the replacement of the Standard Industrial Classification (SIC) system. The standardization of the nomenclature was contributed by the U. S. Economic Classification Policy Committee (EPCP), Statistics Canada, and Mexico's Instituto Nacional de Estadistica y Geografia, and this nomenclature allows detailed and high level comparability among business types. Ring buffers of 2 km each were generated up to a radius of 34 km. This originated into seventeen ring buffers measuring the distance from the Central Business District (CBD). While Mumbai considers several in literature, and no consensus exists, we based our CBD on the oldest (pre-1920) business locations, approximating through a point density map, the centroid locating the precise location of a possible center for a CBD at the beginning of business activity in Mumbai. The spatial analysis prompted to understand the diversity of business type. To achieve this Shannon-Weiner Species Diversity Index was used (Equation 3). To achieve this, the total number of different types was gathered and summed to its proportion for each specific ring buffer. This allowed for a spatial explicit assessment of business diversity in the Greater Mumbai Area considering that:

(3) $$H^{\prime }=\;-\sum_{i=1}^{b}p_{i}ln\;p_{i}$$

where $H^{\prime }$consists of the species diversity index and b is the number of businesses and $\;p_{i}$ is the proportion of businesses of each type belonging to the i species of the total number per each spatial interval.

### 4.1 Self-organizing map

The SOM is an unsupervised Artificial Neural Network, which can be seen as a clustering technique based on the classical vector quantization. The SOM is loosely inspired in what it is thought to be the mechanisms of the human brain, based on the idea that different parts of the brain react, or get activated, by different stimuli. In other words, in the SOM, like in the human brain, certain subgroups of neural units respond selectively to specific sensory stimuli (Kohonen, 2013). SOM's allow for the display of high-dimensional data into a one, two or three-dimensional topology preserving map. Preserving the topological relations means that the components that are similar will tend to appear near each other in the one, two or three-dimensional representation, usually called the output space. Considering this ability to meaningfully map high-dimensional vectors, SOM's have been widely used to extract and visualize the essential structures and characteristics in datasets, through a topology preserving map, which results from an unsupervised learning process (Kaski and Kohonen 1998) (Kaski and Lagus 1996). The most significant advantage of using SOM's is related with the ability to preserve the topological relation between high-dimensional vectors in a one, two or three-dimensional map. The SOM can be seen as an effective tool for data compression and visualization, allowing for the identification of groups of similar vectors, while distinguishing different ones. The resulting topological map can be seen as a representation of the latent structures of the dataset, and it can be valuable in many circumstances, such as clustering, semi-supervised classification, visualization and data reduction. The SOM has been a very popular tool, with a wide range of applications, and its usefulness has been widened through the introduction of numerous variants for specific problems and domains. This is the case of the geospatial domain, where many different extensions have been proposed, ranging from the creation of cartograms (Henriques et al., 2009) to the design of homogeneous regions (Bacao et al., 2004) or the visualization of demographic trajectories (Skupin and Hagelman 2005), just to mention a few.

There are two main concepts to understand the SOM, the input space and the output space. The input space is the original space of the data vectors, and the output space is the n-dimensional (usually 2-dimensional) grid of units, also called neurons, where the data vectors will be mapped. In order to preserve the topological relations present in the input space, the SOM will map data vectors which are closed in the input space into the same or close units in the output space. It is important to note that each unit has the same number of weights as the data vectors and can be seen as a vector in the same space of the data vectors. During training (i.e. the creation of the SOM) each data vector is presented to the SOM and the closest unit is defined as the best matching unit, resulting in the mapping of the data vector onto best matching unit. During training all the data vectors are presented multiple times to the SOM, and through this iterative training process the data vectors, which are close in the input space, will be mapped to units which are close in the output space. The training of a SOM can be seen as a process of density estimation, where the units of the SOM are positioned in order to represent the density of the data vectors distribution. By the end of the training process the units, typically in much smaller number than the data vectors, can be seen as the representatives of the data vectors, mimicking the distribution density over all the input space. For a more detailed explanation of the SOM algorithm the reader is referred to Kohonen (2013).

In the context of this study the SOM can be a valuable tool to improve the understanding of the manifold structure of the data and the identification of the clusters present in the data. In this process of exploring the data will be using the output space of the SOM to understand which are the main groups of companies that can be identified.

## 5 Discussion

**Figure 4:**
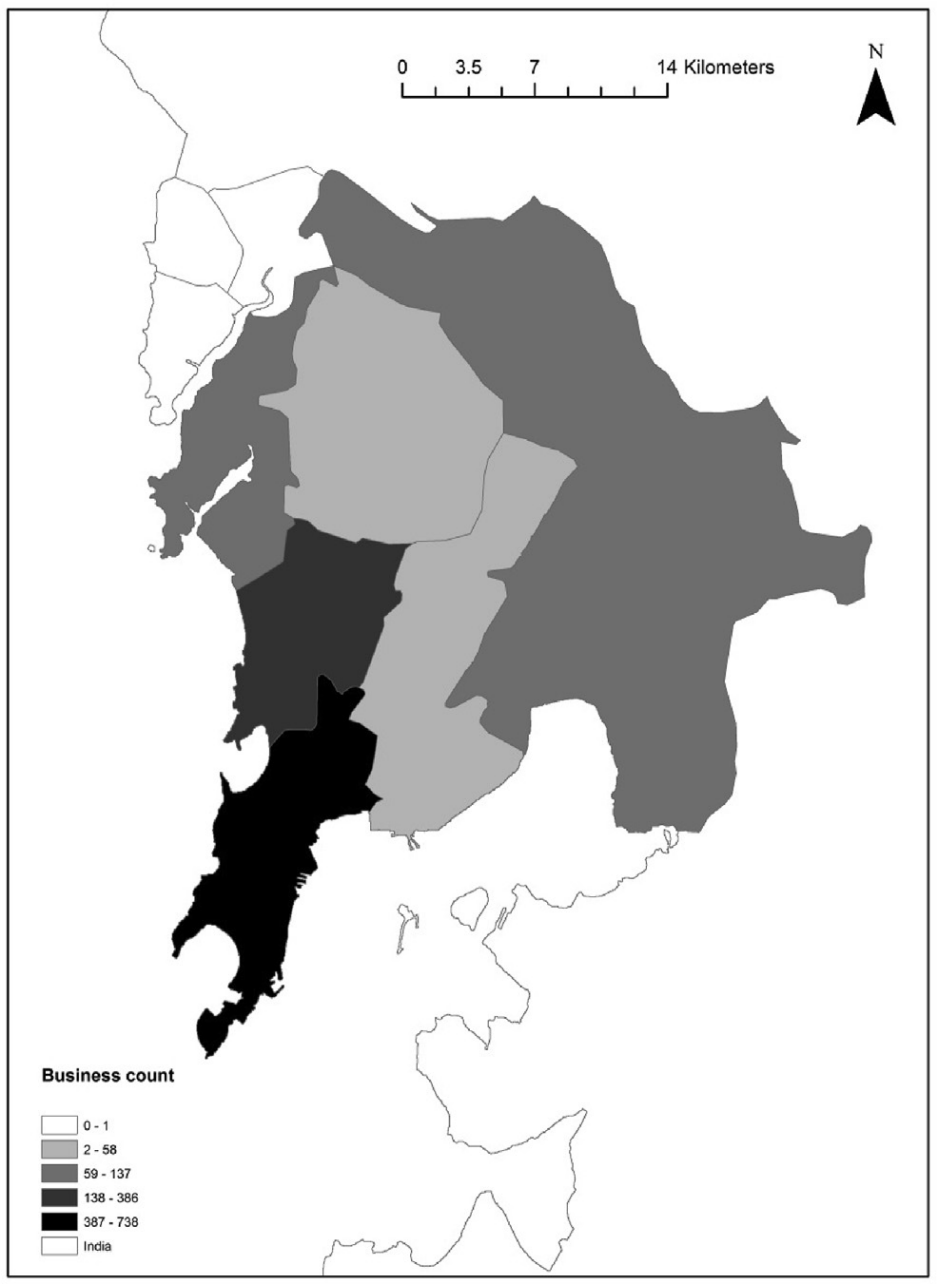
Location of businesses in the Greater Mumbai Area.

The adaptation of this nomenclature to India allowed to have an assessment of Mumbai's distribution of businesses (Figure 4).

**Figure 5:**
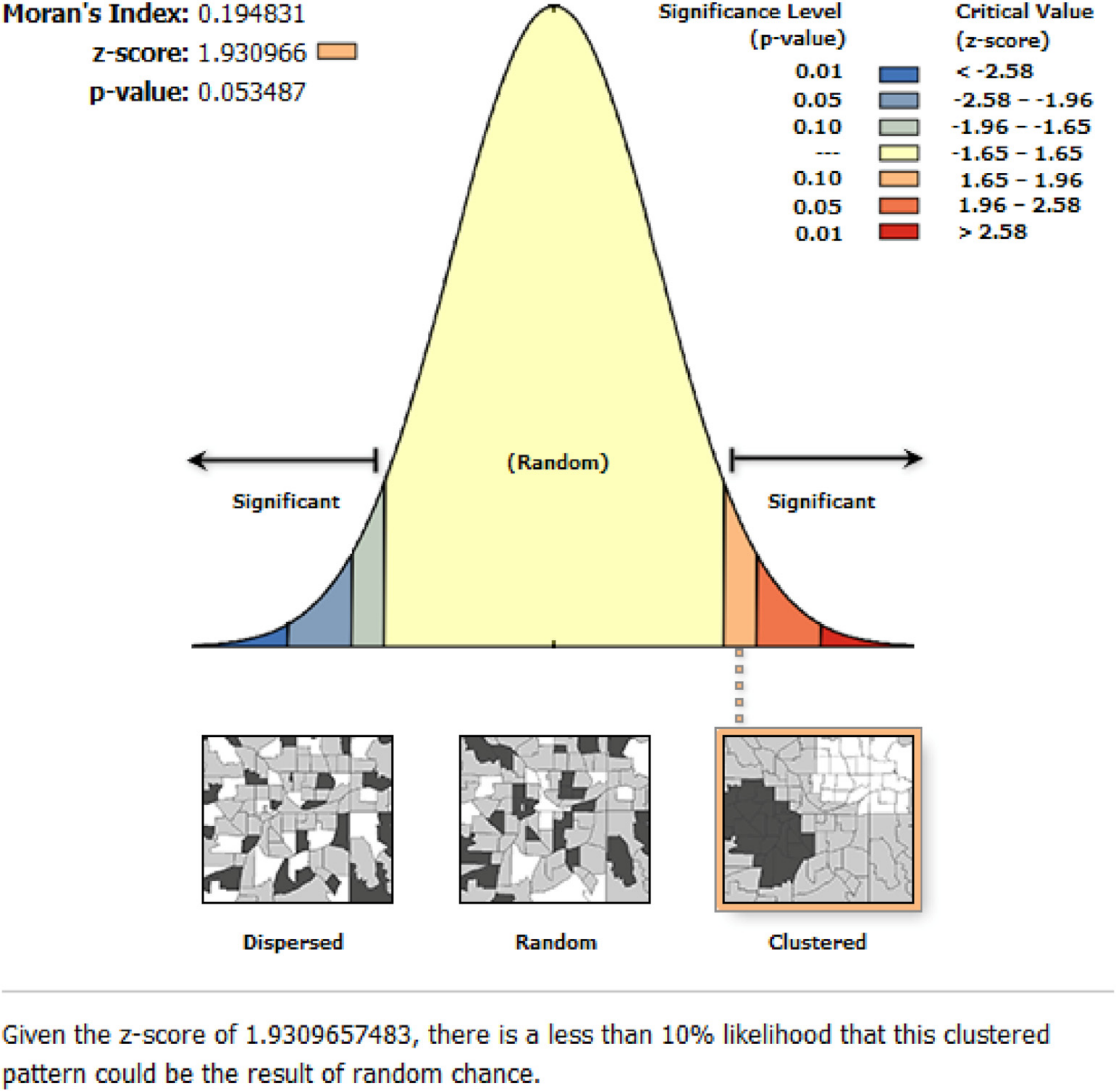
Moran's I for businesses in the Greater Mumbai Area.

Most businesses in the Greater Mumbai Area are located in south Mumbai, close to the financial district of the region. A strong spatial autocorrelation exists at a global level suggesting a very high Moran's I index (Figure 5).

This clustering pattern is a result of the very high spatial autocorrelation found in south India, suggesting that spatial explicit models apply in the context of the Greater Mumbai Area. While businesses in the Greater Mumbai Area do cluster spatially, the effect of diversity of business types must be clearly understood as to assess a spatial landscape of business opportunities. Our integrated approach provided an assessment of the Shannon-Weiner's Diversity Index (Table 2).

**Table 2:** Shannon-Weiner's Diversity Index (SWDI) per distance.

Distance Band	Shannon-Weiner Diversity Index
0 to 2 km	2.823083376
2 to 4 km	3.569255762
4 to 6 km	3.680831313
6 to 8 km	3.065046992
8 to 10 km	2.793656784
10 to 12 km	3.13436171
12 to 14 km	3.528428868
14 to 16 km	3.498295209
16 to 18 km	3.424681375
18 to 20 km	3.245568973
20 to 22 km	2.958113948
22 to 24 km	3.101369337
24 to 26 km	2.748341232
26 to 28 km	2.604187802
28 to 30 km	2.067975912
30 to 32 km	2.853177213
32 to 34 km	1.609437912

**Figure 6:**
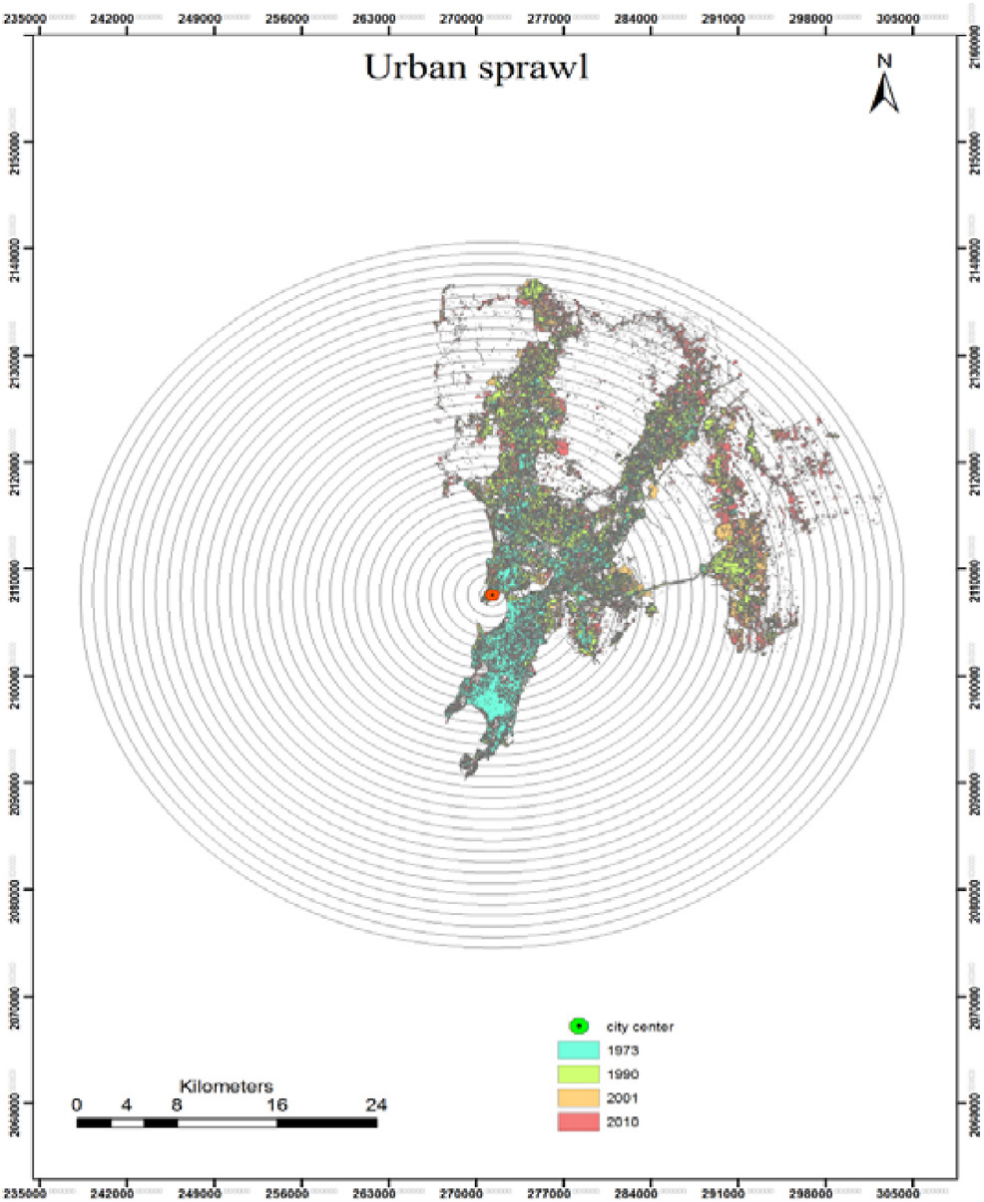
Urban extent for measuring urban sprawl.

A closer analysis of the SWDI allowed establishing a pattern relating that business diversity functions within the First Law of Geography. Diversity tends to decrease over spatial distance but not proportionally to spatial-autocorrelation- A joint assessment with urbanization patterns over the Greater Mumbai Area in a spatio-temporal examination of Mumbai's extent. Understanding the dynamic phenomenon such as urban sprawl over the space and time requires having urban extend. After extracting urban extend over the four-time series, and landscape metrics of Shannon's entropy has been applied for spatial and temporal analysis of urban sprawl to identify the extent and growth pattern of the region (Figure 6).

Due to the high rate of migration and population growth in Mumbai, the sprawl of the city was expected, and Mumbai can in terms of business location vis-à-vis urbanisation, be considered a polycentric city. The amount of built up area was calculated for each buffer zone separately using similar buffers as proposed in the business analysis, these zones, were thus measured through Shannon equation so as to quantify urban sprawl (closer to zero more concentrated, closer to one more dispersed). Table 3 illustrates the resulting dispersion pattern of the Mumbai Metropolitan Region. Mumbai is experiencing sprawl, particularly in the north and eastern parts. For a megacity such as Mumbai, such development is of great concern, as results show an inevitable tendency of urban growth fostering, urban sprawl exists, that not necessarily interacts with the regions that pose most diversified economic growth or correspond to business hubs.

**Table 3:** Multi-temporal changes in Shannon's Entropy.

Selected year	Value of entropy	Difference	Increase (%)
1973	.810	Base year	Base year
1990–1973	.849	.039	4,60%
2001–1990	.865	.016	1,90%
2010–2001	.893	.027	3,10%

The usage of spatial analysis to geocode businesses and assess therefore underlying patterns, is a unique methodology to infer additional information on spatio-temporal business clusters, but also to better understand the business ecology and its intricate relation to space, which often is not fully assessed at the regional level. The availability of the urban footprint from the German Aerospace Center (DLR), as discussed in Taubenböck et al. (2009), permitted to have a regional footprint of the Mumbai Metropolitan Region (MMR) which allowed to overlay the available land use datasets to a temporal proxy of the business landscape. A first very generic visual interpolation, allowed to understand that urban and business locations are intrinsically related, a better understanding on the quantification of this relation has not been explored, and the availability of tools found in landscape metrics such as understanding the compactness of urban density within a certain restricted geometrical area could allow to better represent the relation of urban density in regards to business concentration from the central business district. This was achieved by generating multiple ring buffers equidistant of 1 km from the CBD. In the case of the cluster analysis resulting from the application of the SOM, the results provide important insights for understanding the relationships between business density and their proximity to the central business district (CBD) in Mumbai. CBD's are an important component of major urban centres throughout the world and they often are significant and extensive facilitators of money flows, employment, infrastructure development and concentration of values (Taubenböck et al., 2013). In Mumbai, rapid urban expansion has been occurring over the past several decades and is expected to continue to increase in future decades. Projections of future expansion suggest that expansion will coincide with existing infrastructure, including existing transportation networks (Moghadam and Helbich, 2013). The above points illustrate the importance of measuring the business landscape of Mumbai and developing an understanding of the existing spatial relationships between business density and proximity in this city.

**Figure 7:**
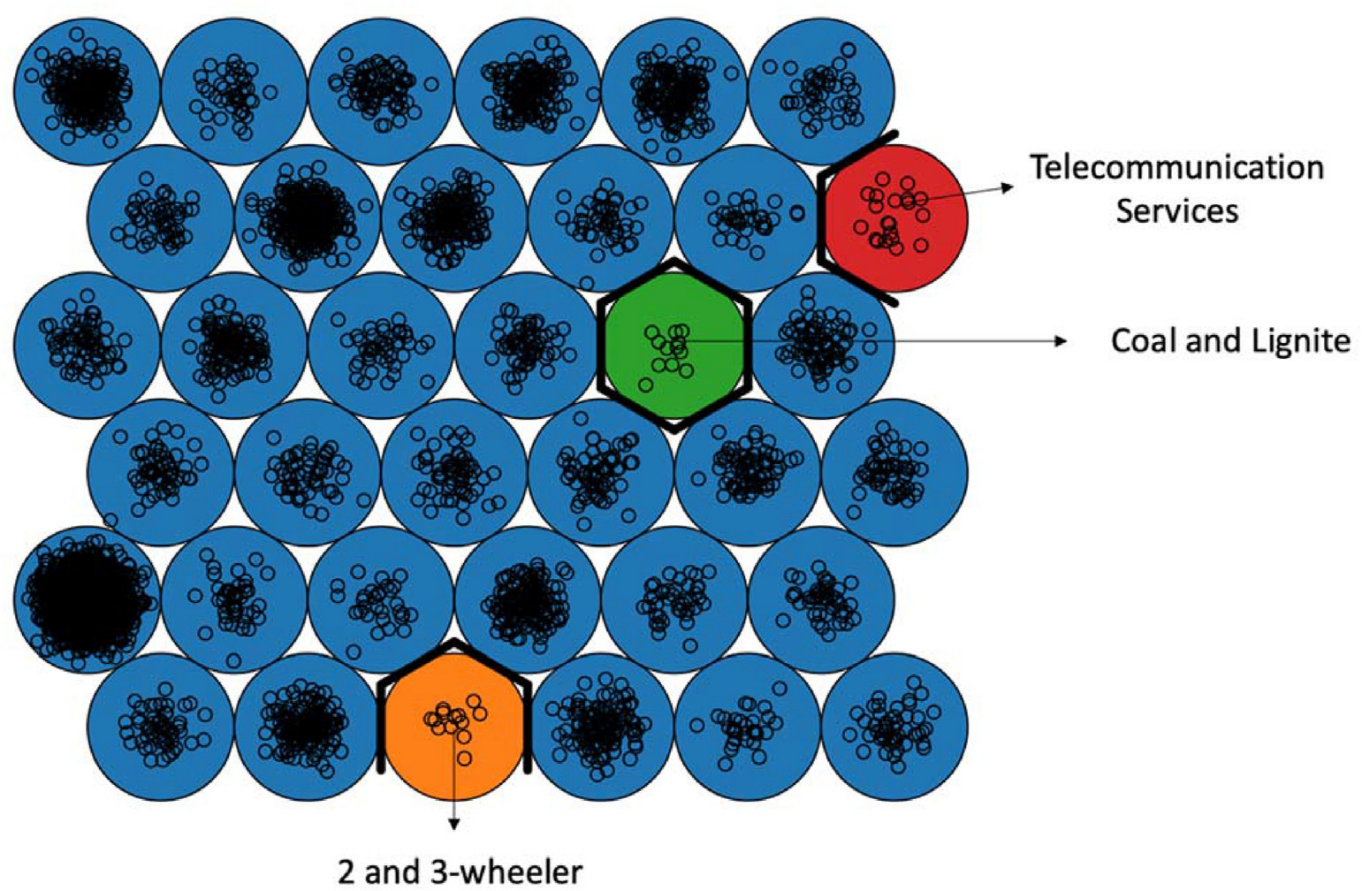
SOM clusters.

A detailed analysis of the Self-Organizing Map results reveal that the general distribution is fairly stable, suggesting that similar industries locate within precise geographical clusters, nevertheless there are 3 main outlier units, shown in red, green and orange on Figure 7. The red unit represents 20 companies operating in the telecommunications services cluster. These are relatively new companies, from the 1990's and are located close to the CBD. On a completely different note, the green unit represents coal and lignite companies (13 companies) which, contrary to the previous example, are older companies, from the 70's, and located far away from the CBD. Finally, the orange unit represents a specialized cluster of 12 companies working on 2 and 3 wheeler, located closely to the automobile ancillary cluster.

## 6 Conclusion

Growing megacities such as Mumbai have shown increasing urbanization processes largely brought by population density and incremental economic activity. Mumbai's metropolitan area's core has been one of the fastest-growing regions globally, creating a nexus of urban sprawl and cumulative business activity, leading to India's urban commercial industry's diversified tradition merged with the effects of population dynamics.

With the rapid change of population dynamics and economic activity, the fragmentation of previous urban landscapes has become a detrimental concern as Mumbai's economy grew over the last two decades (Young and Jarvis, 2008). This paper suggested a complementary approach to abridge the spatial narrative of businesses, and the role of territorial proximity in urban cores, a growing issue in developing countries. Concerning south Asian megacities such as Mumbai, there is significant evidence that infrastructures have to be managed depending on the folio of businesses and commercial activity within the surrounding landscapes.

This is especially the case considering the geodemographic transitions of populations, where urban sprawl and the disparity between income and regional impacts must be considered carefully and find sustenance between functional business activity rhetoric. Commercial activity at its core, maybe a positive driver for growing urban regions but requires a careful strategic plan that intertwines the dynamics of population, landscape, and the possibility of defining determinants of economic activity in an agenda of urban processes. The integration of spatially-explicit tools as discussed in this paper utilizing SOM, offers significant insights for a combined approach to understand the geographical space. These regional tools also bring functionality within a paradigm of urbanization and clusters of commercial land. Urbanization plays a key role for future sustainability within the urban nexus and abridges the financial district throughout the growing conclave or Mumbai's business activity.

## Declarations

### Author contribution statement

Eric Vaz, Bruno Damásio, Fernando Bação, Mahender Kotha, Elissa Penfound: Conceived and designed the experiments; Performed the experiments; Analyzed and interpreted the data; Contributed reagents, materials, analysis tools or data; Wrote the paper.

Shailendra Kumar Rai: Contributed reagents, materials, analysis tools or data; Wrote the paper.

### Funding statement

This research did not receive any specific grant from funding agencies in the public, commercial, or not-for-profit sectors.

### Data availability statement

Data will be made available on request.

### Declaration of interests statement

The authors declare no conflict of interest.

### Additional information

No additional information is available for this paper.

## Footnotes

1 https://github.com/geopy/geopy.
